# Does imitating the “hand-gesture dance” on social media reduce anxiety? A two-wave cross-lagged panel analysis on digital embodied practices

**DOI:** 10.3389/fpsyg.2026.1934635

**Published:** 2026-10-14

**Authors:** Yufei Mao, Dina Tu, Yun Feng

**Affiliations:** 1 College of Music, Shanghai Normal University, Shanghai, China; 2 School of Communication, Soochow University, Suzhou, Jiangsu, China; 3 School of Journalism and Information Communication, Huazhong University of Science and Technology, Wuhan, Hubei, China

**Keywords:** anxiety, cross-lagged panel model, hand-gesture dance, interpersonal sensitivity, self-surveillance, short-video

## Abstract

**Introduction:**

This study examines the longitudinal associations among hand-gesture dance participation (HGDP), self-surveillance (SS), interpersonal sensitivity (IS), and anxiety (ANX) among Chinese adults.

**Methods:**

Using a two-wave longitudinal survey with a 1-month interval, data were collected from a Chinese sample at Time 1 (*n* = 1,500) and Time 2 (*n* = 1,003). Six competing two-wave traditional cross-lagged panel models (CLPMs) were tested in Mplus 8.3 to examine temporal associations among the four constructs.

**Results:**

The fully crossed model (M6) showed the best fit among the alternative models based on the overall pattern of fit indices and information criteria, with acceptable absolute fit. Results showed that HGDP at T1 was negatively associated with anxiety at T2 (β = −0.128, *p* < 0.001) and interpersonal sensitivity at T2 (β = −0.158, *p* < 0.001), and showed a marginally significant negative association with self-surveillance at T2 (β = −0.051, *p* = 0.050). The variables also exhibited multidirectional cross-lagged associations rather than a single unidirectional pathway, with self-surveillance, interpersonal sensitivity, and anxiety at T1 each predicting one or more subsequent constructs at T2.

**Discussion:**

These findings indicate that greater hand-gesture dance participation was temporally associated with lower subsequent anxiety and interpersonal sensitivity and, more tentatively, with lower self-surveillance. Because the HGDP index encompasses different modes of engagement, including viewing, private imitation, and recording/posting, these associations should be interpreted as associations with participation intensity rather than as evidence of a specific causal effect of embodied movement. The study highlights the importance of distinguishing heterogeneous forms of social media participation when examining their psychological correlates over time.

## Introduction

Anxiety is a prevalent negative psychological state in contemporary society and has become a major factor affecting the mental health of young people (Tan et al., 2023). Epidemiological studies indicate that anxiety disorders are among the most common mental disorders in China. The annual prevalence of anxiety disorders among Chinese adults is 5.0%, while the lifetime prevalence reaches 7.6%, exceeding that of depressive disorders (Huang et al., 2019). As the pace of social life accelerates, life pressures accumulate, and the information environment becomes increasingly complex; the psychological burden experienced by young people continues to intensify, leading to a marked increase in anxiety levels. A study published in The British Journal of Psychiatry reports that the number of individuals with anxiety disorders in China increased from 40.5 million in 1990 to 53.1 million in 2021, representing a growth of 31.2% (Tian et al., 2026). Anxiety not only impairs emotional regulation and cognitive functioning but also disrupts multiple aspects of daily life, including learning, work, and social interaction, thereby exerting broad consequences for public mental health. Against this background, identifying effective approaches to anxiety intervention for young people has become a central concern in contemporary research.

Previous studies show that physical activity can alleviate anxiety by regulating neurotransmitters and improving psychological functioning (DeBoer et al., 2012). Embodied forms of physical activity, such as dance and exercise (Koch et al., 2019; Singh et al., 2023), demonstrate significant anxiety-reducing effects and contribute positively to mental health. Social engagement also plays an important role in psychological wellbeing. Increasing social participation and reducing behavioral avoidance enhances individuals' sense of self-worth and self-identity, thereby reducing anxiety levels (Lee and Yeghiazarian, 2021). In addition, support received from communities and social groups effectively mitigates anxiety (Ciułkowicz et al., 2022). These findings suggest that both embodied behavior and social interaction constitute important pathways for alleviating anxiety.

Although hand-gesture dance has attracted substantial attention among young people (Wang et al., 2022), its influence on mental health outcomes, particularly anxiety, remains underexplored. As a novel form of dance practice emerging within digital media environments, hand-gesture dance has not yet been examined through systematic empirical research regarding its psychological effects. With the widespread adoption of social media, digital environments have emerged as distinctive contexts for interaction, reshaping patterns of cognition, emotion, and behavior while providing new channels for the expression and dissemination of traditional physical activities and social interactions (McFarland and Ployhart, 2015). Platforms such as short-video applications and live-streaming services enable bodily participation to transcend physical constraints and be widely disseminated in symbolic and digital forms (Tao et al., 2026). Within this context, hand-gesture dance, a form of digital embodied practice that has gained popularity on short-video platforms in recent years, exhibits a deep integration of physical activity and social interaction. It retains the exercise-related functions of traditional dance while incorporating features of digital social engagement. Existing research indicates that exposure to dance-related short-video content influences women's body satisfaction (Joiner et al., 2023). Moreover, dance, as a representative form of physical activity, has been widely demonstrated to generate positive effects on mental health (Karkou et al., 2019).

Therefore, this study examines the temporal associations among hand-gesture dance participation, self-surveillance, interpersonal sensitivity, and anxiety among Chinese adults using a two-wave longitudinal design.

## Literature review

### Social media participation and mental health: dual effects

Existing research in communication and mental health has identified social media participation as an important factor influencing individuals' psychological wellbeing (Godard and Holtzman, 2024). A substantial body of research examines dimensions such as usage duration and interaction patterns, revealing complex relationships between social media participation and psychological outcomes, including anxiety, depression, and self-esteem. Recent meta-analytic evidence further suggests that the psychological consequences of social media use depend substantially on the nature and quality of engagement rather than on exposure alone. A meta-analysis of 78 studies found that social media communication, active use, and positive online experiences were positively associated with several indicators of wellbeing, whereas problematic use and social comparison were negatively associated with positive wellbeing (Marciano et al., 2024). Similarly, a recent scoping review of reviews found that problematic and passive social media use were more consistently associated with depression, anxiety, and loneliness, while purposeful use that facilitates social support and reduces social isolation may yield beneficial psychological outcomes (Agyapong-Opoku et al., 2025). Studies indicate that longer social media use and more frequent platform visits are associated with higher levels of depression, anxiety, and loneliness (Corke et al., 2025). Other studies further demonstrate that different patterns of social media use produce distinct psychological consequences. For example, browsing and interaction behaviors on Instagram are negatively associated with loneliness (Verduyn et al., 2017). On the one hand, social media provides channels for emotional expression, information acquisition, and social support, which helps reduce stress and strengthens social connectedness (O'Reilly et al., 2023). This positive potential is also supported by evidence showing that direct communication and reciprocal self-disclosure through social media can alleviate loneliness and stress, particularly when online interactions provide meaningful interpersonal connection (Marciano et al., 2022). On the other hand, its highly visualized and evaluation-oriented interaction mechanisms may facilitate social comparison, the accumulation of negative emotions, and difficulties in self-identity formation (Wirtz et al., 2021; Irmer and Schmiedek, 2023; Avci et al., 2025). Consequently, social media participation cannot be characterized as either uniformly beneficial or harmful. Rather, its psychological effects vary across contexts and modes of use. Importantly, these effects also differ according to how individuals participate. Passive viewing primarily involves exposure to others' content, whereas active participation, such as commenting, interacting, or creating content, involves greater behavioral and interpersonal engagement. Existing findings therefore suggest that treating social media use as a single construct may obscure meaningful differences between participation modes. Moreover, much of the existing evidence is based on cross-sectional associations, which limits conclusions about the temporal direction of these relationships.

### Media characteristics and dual attributes of short-video hand-gesture dance

With the evolution of media technologies, short-video platforms have become a central component of the contemporary social media ecosystem (Zheng, 2023). Short-video participation is generally defined as a range of behavioral practices on short-video platforms, including content viewing, liking, commenting, creating, and sharing (Omar and Dequan, 2020). At its core, it represents a continuous process of user engagement shaped jointly by algorithmic recommendations and social interaction mechanisms. Compared with traditional text- and image-based social media, short-video platforms are more likely to attract user participation because of their distinctive social features and low technological barriers (Zhang et al., 2019). As noted earlier, the effects of social media engagement on individual psychology are neither unidirectional nor stable but instead vary significantly across different usage contexts. Research by Yang et al. (2025) suggests that short-video participation may provide emotional relief through entertainment and immediate gratification. However, Zhang et al. (2024) show that it may also increase anxiety and psychological stress through information overload, loss of time control, and addictive usage patterns.

Alongside increasing attention to media participation, the psychological implications of embodied behavior have also gained scholarly interest. Embodiment theory emphasizes that the body is not merely a vehicle for behavior but also a fundamental component of cognition and emotion (Csordas, 1995). This perspective suggests that bodily engagement may shape psychological experiences through the interaction between physical action and social context. In the context of short-video platforms, where bodily performances can be recorded, viewed, and evaluated by others, the psychological meaning of embodied behavior may extend beyond the immediate experience of movement itself. Collective bodily practices can strengthen individuals' sense of belonging and social connectedness through synchrony and interaction, thereby generating positive mental health outcomes. Tunçgenç and Cohen (2018) find that synchronized movement strengthens social connectedness and reduces intergroup bias. Embodied activities such as dance and physical exercise are widely recognized for their positive effects on emotional regulation, stress reduction, and psychological recovery (Ritter and Low, 1996). Nevertheless, when bodily behavior is situated within a context of public display, its meanings and consequences may change. This distinction is particularly important because physical activity and appearance-oriented self-presentation represent related but conceptually different forms of bodily engagement. Physical activity primarily involves movement and its potential physiological and psychological benefits, whereas appearance-oriented self-presentation places greater emphasis on how the body is displayed, evaluated, and perceived by others. Thus, evidence supporting the benefits of dance or physical exercise cannot necessarily be generalized to embodied practices performed for public visibility on social media. In social media environments, bodily presentation is often accompanied by mechanisms of evaluation, comparison, and observation (Sarda et al., 2025). Such reflective cognition from the perspective of others may further stimulate social comparison and self-doubt (Jiang and Ngien, 2020), thereby producing complex psychological outcomes.

Against this backdrop, hand-gesture dance has emerged as a representative behavioral form on short-video platforms and warrants scholarly attention. Research suggests that content on TikTok is characterized by a high degree of imitability and replicability, enabling ordinary users to reproduce and recreate existing content while reducing barriers to content production (Zulli and Zulli, 2022). Building upon these platform characteristics, hand-gesture dance further operationalizes this logic of imitability. Klug (2020) demonstrates that TikTok dances are typically repetitive, easy to learn, and accessible to a broad audience. Hand-gesture dance, which centers on coordinated hand movements, is characterized by clear rhythmic patterns and simplified movement structures. From the perspective of participation mechanisms, users reproduce content through participation in dance challenges, a process fundamentally based on imitation and creative adaptation (Ng et al., 2021). Individuals may engage with hand-gesture dance in multiple ways. They can consume content as viewers, imitate movements privately for entertainment, or upload their own performances for public display and social interaction. Consequently, the dance not only inherits the core characteristics of short-video content but also combines media participation with embodied practice. Through its distinctive movement structures and dissemination mechanisms, it constitutes a unique form of media practice that differs from traditional dance.

Previous research has documented the positive contributions of dance to mental health from multiple perspectives. Empirical findings indicate that dance participation is significantly associated with higher levels of mindfulness and life satisfaction and serves as a strong predictor of subjective wellbeing (Muro and Artero, 2017). Intervention studies further demonstrate that sports dance training improves psychological wellbeing among university students, enhances resilience to stress, and reduces psychological difficulties (Xu and Liu, 2026). Review-based evidence suggests that line dancing improves physical functioning while reducing symptoms of depression and anxiety and promoting social interaction and community engagement (Cleary et al., 2025). Similarly, a systematic review focusing on older adults reports that square dance interventions generate positive outcomes in emotional wellbeing, cognitive functioning, and social participation (Ou et al., 2022). At the experiential level, dance provides opportunities for emotional expression and psychological adjustment, enabling participants to experience enjoyment, strengthen self-confidence, and establish social connections through interaction (Wildeman et al., 2025). However, longitudinal evidence from Chinese adolescents indicates that different types of physical activity exert distinct effects on social skills and antisocial behavior, with dance participation showing a negative association with social skills development (Yang et al., 2024).

Despite the growing body of research on embodied behavior and dance practice, existing studies are predominantly conducted in offline settings or intervention-oriented experimental contexts (Moratelli et al., 2023). Most attention has been devoted to dance and movement therapy, physical activity interventions, and mechanisms of group interaction. In contrast, relatively little research examines how embodied behavior is embedded within digital media environments and how such contexts shape its psychological consequences. Particularly on highly mediated and visualized short-video platforms, bodily behavior functions not only as a process of personal experience but also as an object of observation, evaluation, and dissemination. As a result, the communicative context surrounding embodied behavior differs substantially from that of offline environments. Consequently, conclusions derived from offline or non-mediated contexts cannot be directly generalized to practices occurring on short-video platforms. This limitation also reflects a difference in the context of participation. Offline dance generally emphasizes movement, exercise, emotional expression, or interpersonal interaction, whereas hand-gesture dance on short-video platforms may additionally involve recording, uploading, audience exposure, and feedback. The same bodily movement may therefore carry different psychological meanings depending on whether it is performed privately, shared with others, or publicly displayed online.

Given the current state of research, the longitudinal psychological correlates of hand-gesture dance participation remain insufficiently understood. As a behavioral form that simultaneously embodies physical practice and social media participation, hand-gesture dance may extend the positive effects of traditional dance on emotional regulation and social connectedness. At the same time, its integration into the display-oriented logic and interaction mechanisms of short-video platforms may introduce additional pathways involving social comparison and self-presentation pressures. Within this context, shaped by the intersection of embodied practice and digital media participation, it remains unclear how hand-gesture dance participation is temporally associated with anxiety and related psychological constructs. Empirical evidence addressing this question is still limited.

### The bidirectional relationship between hand-gesture dance participation and self-surveillance

According to Objectification Theory, individuals who are continuously exposed to contexts characterized by visibility and evaluation are likely to internalize themselves as objects to be observed and judged by others (Fredrickson and Roberts, 1997). This process is particularly salient when individuals' bodily performances become visible to others, as attention may shift from the experience of performing an activity to how the body and its performance are perceived and evaluated. In social media environments, public display behaviors and frequent interaction mechanisms may therefore reinforce self-surveillance by directing individuals' attention toward their own appearance and performance. As a result, they increasingly monitor their appearance and performance from an observer's perspective, developing a tendency toward self-surveillance (Fredrickson and Roberts, 1997). Within social media environments, public display behaviors and frequent interaction mechanisms further reinforce this process. However, the degree of visibility varies substantially across participation modes. Private imitation allows individuals to engage in the embodied activity without necessarily exposing their performance to an audience, whereas public posting introduces additional opportunities for observation, feedback, and social evaluation. This distinction is theoretically relevant because self-surveillance is more directly implicated when individuals anticipate or receive evaluation of their visible bodily performance. Through content creation and feedback reception, users continuously adjust their self-presentation strategies, thereby increasing attention to and reflection on their external appearance and performance. Previous research suggests that social media use influences individuals' levels of self-objectification and intensifies their monitoring and concern regarding outward appearance and self-presentation (Sarda et al., 2025). At the same time, studies indicate that adolescents' existing levels of self-objectification predict subsequent increases in self-presentational behaviors such as selfie posting (Wang et al., 2021). Appearance-related comments further strengthen state self-objectification, while higher levels of self-objectification increase individuals' willingness to continue sharing self-presentational content on social media (Vendemia and DeAndrea, 2021). These findings suggest that self-surveillance may not only emerge as a consequence of self-presentational behavior but may also motivate individuals to engage more frequently in such practices.

The existing literature indicates that the self-surveillance process, which is rooted in self-objectification, functions not only as an outcome of self-presentational behavior but also as an antecedent that continuously drives participation in such activities. From this perspective, hand-gesture dance constitutes a form of embodied self-presentational behavior on short-video platforms. During participation, individuals are required to pay continuous attention to their bodily performance, while the social media environment in which the dance is embedded further amplifies feedback from others and shapes self-perception. Consequently, self-surveillance and hand-gesture dance participation may exert reciprocal influences on one another. Based on this reasoning, the following hypothesis is proposed:

**Hypothesis 1**. *There are reciprocal temporal associations between hand-gesture dance participation on short-video platforms and self-surveillance*.

### The bidirectional relationship between hand-gesture dance participation and interpersonal sensitivity

With respect to interpersonal sensitivity, Social Comparison Theory proposes that individuals naturally engage in comparison when exposed to information about others' performance and subsequently evaluate their own abilities and social standing based on such comparisons (Festinger, 1954). Such comparison processes become particularly salient in social media environments, where individuals are repeatedly exposed to others' selectively presented performances while receiving visible social feedback. Repeated comparison with others may consequently heighten attention to social evaluation and perceived differences between oneself and others, providing a pathway through which media participation may be associated with interpersonal sensitivity. Commenting and being commented upon constitute fundamental manifestations of social media interactivity (Lee et al., 2015). Furthermore, visible feedback indicators such as likes and comments provide readily accessible benchmarks for comparison, reinforcing both upward and downward comparison processes (Sánchez-Hernández et al., 2022).

Within such environments, individuals become increasingly attentive to others' opinions and are continuously influenced by evaluative cues during platform use. Previous research indicates that concerns about negative evaluation and the need for social approval in social media contexts are closely associated with social comparison processes and significantly influence users' online behaviors (Nor et al., 2025). At the same time, frequent social media use increases opportunities for exposure and self-presentation. Such exposure stimulates social comparison, while the outcomes of these comparisons are immediately reinforced through platform-based interaction mechanisms, which in turn influence subsequent usage behaviors (Mehdizadeh, 2010). Research further demonstrates that self-presentation on social media affects individuals' perceptions of self and social relationships through social comparison processes, particularly influencing interpersonal sensitivity (Shin et al., 2017). As one of the core dimensions measured by the SCL-90, interpersonal sensitivity inherently contains a strong social comparison component and is characterized by feelings of inferiority and discomfort in comparison with others (Timman and Arrindell, 2022). It is also important to distinguish interpersonal sensitivity from broader forms of anxiety or general psychological distress. Interpersonal sensitivity specifically concerns heightened concern about interpersonal evaluation, rejection, inferiority, and discomfort in social relationships, making it particularly relevant to social-media environments characterized by visibility and comparison. This construct-specific perspective provides a more precise basis for examining how evaluative participation may relate to interpersonal sensitivity. Consequently, this construct is found to be highly susceptible to individuals' self-presentational experiences within environments characterized by observation and evaluation from others.

Continuous engagement in self-presentational social behaviors may increase individuals' reliance on external evaluations when adjusting their modes of self-expression. Conversely, individuals with higher levels of interpersonal sensitivity may be more likely to modify their participation behaviors according to others' feedback. This dynamic suggests a reciprocal process between psychological dispositions and behavioral engagement. Therefore, within the context of hand-gesture dance, a practice that emphasizes both public display and content dissemination, interpersonal sensitivity and participation behavior may reinforce one another. Accordingly, the following hypothesis is proposed:

**Hypothesis 2**. *There are reciprocal temporal associations between hand-gesture dance participation on short-video platforms and interpersonal sensitivity*.

### The bidirectional relationship between hand-gesture dance participation and anxiety

Anxiety, as a typical psychological response to persistent evaluative pressure and uncertain feedback, is widely recognized in social media research as being closely associated with frequent interaction, social comparison, and dependence on external feedback (Valkenburg et al., 2022). Existing studies demonstrate a consistent relationship between social media use and mental health outcomes, with negative emotional states such as anxiety and depression emerging across various usage contexts. Behaviors including multitasking, emotional investment, appearance-oriented activities, and passive browsing are all associated with poorer psychological wellbeing (Prasad et al., 2023). In addition, the intensity of social media use is consistently linked to anxiety levels, with longer usage durations predicting more severe anxiety symptoms (Vannucci et al., 2017). Building upon these findings, subsequent research suggests that the relationship between social media use and anxiety is not unidirectional. Individuals experiencing anxiety may increase their engagement with social media in an attempt to seek relief or distraction, thereby further increasing the frequency and intensity of their platform use (Zsido et al., 2021).

Most previous studies have focused on the potentially negative psychological consequences of self-presentation on social media, particularly negative social comparison and the emotional distress that may result from it. However, self-presentation on social media encompasses diverse behavioral orientations, including active content creation, interactive participation, and passive content consumption (Gao et al., 2023). As discussed earlier, among these various forms of self-presentation, one distinctive form combines physical activity with self-presentation. A substantial body of research demonstrates that physical exercise itself exerts beneficial effects on anxiety reduction and emotional wellbeing through mechanisms such as physiological regulation and emotional release (Ensari et al., 2015; Su et al., 2026). Nevertheless, relatively little attention has been devoted to the psychological consequences of exercise-based self-presentation on social media, a behavioral form that may simultaneously generate both positive and negative effects.

Moreover, although a large proportion of the literature suggests that social media self-presentation may contribute to anxiety and other negative emotions, some studies indicate that self-presentation can increase social capital and perceived social support, thereby promoting positive emotional experiences (Huang, 2016). Frison and Eggermont (2016) find that adolescents who actively engage in self-presentation through content posting report fewer depressive symptoms than those who primarily consume others' content through passive observation. Furthermore, positive feedback on social media enhances positive affect and social belongingness, which in turn encourages greater engagement in self-presentational behaviors (Brudner et al., 2023).

Taken together, existing findings reveal considerable inconsistency regarding the relationship between social media self-presentation and anxiety. However, this inconsistency should also be interpreted in light of differences in research design. Much of the literature relies on cross-sectional associations, which establish relationships between variables but provide limited evidence regarding temporal ordering or causal direction. Experimental and longitudinal studies offer stronger leverage for examining whether particular forms of participation precede subsequent psychological changes, although such evidence remains comparatively limited for embodied self-presentation on short-video platforms. Although the overall direction of this relationship remains unclear, the literature consistently suggests a significant association between the two constructs. Within this context, hand-gesture dance represents a participatory practice that integrates bodily expression with public self-presentation on short-video platforms. It involves content creation and self-presentation while simultaneously operating within an environment characterized by continuous interaction and feedback. Consequently, participation in hand-gesture dance may be influenced by individuals' emotional states and may, in turn, shape their emotional experiences. Based on this reasoning, the following hypothesis is proposed:

**Hypothesis 3**. *There are reciprocal temporal associations between hand-gesture dance participation and anxiety*.

### The internal cyclical structure between self-surveillance, interpersonal sensitivity, and anxiety

Regarding the structural relationships between the focal variables, Objectification Theory and Social Comparison Theory jointly illuminate the psychological processes through which individuals respond to highly visible social environments. Objectification Theory emphasizes the internalization of an observer's perspective and the resulting monitoring of one's own appearance and performance, whereas Social Comparison Theory emphasizes the evaluation of oneself in relation to others. These processes may operate simultaneously in social media contexts, where individuals are both objects of observation and observers of others' performances. When individuals engage in persistent self-surveillance, their attention to external evaluative cues increases correspondingly (Canvin et al., 2016), which may heighten interpersonal sensitivity. Conversely, elevated interpersonal sensitivity may further encourage individuals to internalize external evaluative standards, thereby intensifying self-surveillance. As a result, a mutually reinforcing feedback mechanism may emerge between these two psychological processes. Such an interactive structure places individuals in a prolonged state of self-monitoring and may generate cumulative effects on the experience of psychological stress.

At the level of psychological outcomes, the relationships between self-surveillance, interpersonal sensitivity, and anxiety also exhibit dynamic characteristics. Persistent self-monitoring amplifies individuals' perceptions of deficiencies in their own performance, thereby increasing anxiety levels (Spurr and Stopa, 2002). In turn, anxiety heightens concern and vigilance regarding personal performance, further strengthening self-surveillance. Meanwhile, the anticipated evaluation and social comparison pressures associated with interpersonal sensitivity may lead individuals to perceive themselves as inferior to others, thereby increasing the risk of anxiety (Lee, 2014). These findings suggest that self-surveillance may be associated with subsequent anxiety indirectly through interpersonal sensitivity, while interpersonal sensitivity may also show a direct temporal association with anxiety. At the same time, anxiety may subsequently be associated with higher levels of both self-surveillance and interpersonal sensitivity.

Accordingly, these three constructs may form an interconnected psychological feedback system. Within this system, self-surveillance, interpersonal sensitivity, and anxiety may exert reciprocal influences on one another. Based on this reasoning, the following hypotheses are proposed:

**Hypothesis 4**. *There are reciprocal temporal associations between self-surveillance and interpersonal sensitivity*.

**Hypothesis 5**. *There are reciprocal temporal associations between self-surveillance and anxiety*.

**Hypothesis 6**. *There are reciprocal temporal associations between interpersonal sensitivity and anxiety*.

Therefore, we propose the following model (See Figure 1).

**Figure 1:**
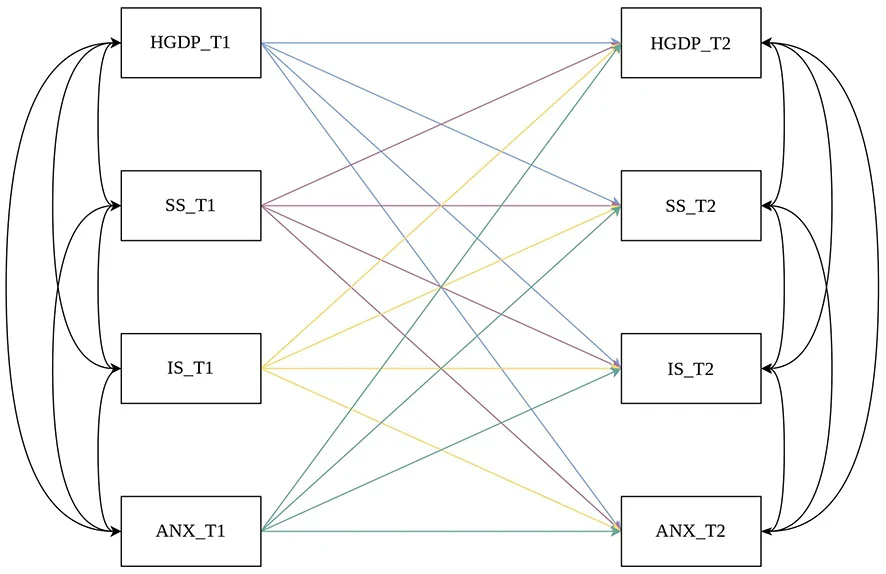
Hypothesized research model.

## Materials and methods

### Participants

This study recruited participants nationwide through the online survey platform Credamo, with participants from 32 provincial-level administrative regions across China, and conducted a two-wave longitudinal survey with a 1-month interval. Eligible participants were Chinese adults aged 18 years or older who were familiar with and had previously watched hand-gesture dance content on social media. The survey included attention-check items, and duplicate responses were screened using IP addresses and platform-level response information. Participants who failed the attention checks or were identified as duplicate responses were excluded. The first wave of data collection (T1) was conducted in October 2025, yielding 1,500 valid responses. The second wave (T2) was conducted in November 2025, yielding 1,003 valid responses. Thus, 1,003 of the 1,500 T1 participants were retained at T2, corresponding to a follow-up retention rate of 66.87%. Participants across the two waves were matched using their Credamo user IDs.

At T1, the full baseline sample (*n* = 1,500) ranged in age from 18 to 65 years, with a mean age of 31.20 years (SD = 7.828). The sample consisted of 33.4% males and 66.6% females. To assess potential attrition bias, participants who completed both waves (*n* = 1,003) were compared with those who dropped out after T1 (*n* = 497) on baseline demographic and study variables. For categorical variables, chi-square tests were conducted and effect sizes were assessed using Cramér's V. For continuous variables, independent-samples *t tests* were conducted and Cohen's d was reported.

No statistically significant differences were observed between completers and dropouts in gender (χ^2^ = 0.487, *p* = 0.485, ϕ = 0.018), education level (χ^2^ = 7.203, *p* = 0.302, V = 0.069), or income (χ^2^ = 8.617, *p* = 0.071, V = 0.076). Although the association between income and attrition approached the conventional significance threshold, its effect size was very small (V = 0.076), indicating that the magnitude of the difference was limited. Among the continuous baseline variables, completers were slightly older than dropouts (M = 32.60, SD = 8.15 vs. M = 30.35, SD = 6.87; *p* < 0.001, Cohen's d = 0.31). Small differences were also observed for self-surveillance (*p* = 0.001, d = 0.19), interpersonal sensitivity (*p* = 0.007, d = 0.15), and anxiety (*p* = 0.015, d = 0.13), whereas hand-gesture-dance participation showed a negligible difference (*p* = 0.390, d = 0.05).

Overall, although several baseline variables differed statistically between completers and dropouts, the corresponding effect sizes were small, with the largest effect observed for age (d = 0.31). These findings suggest that the magnitude of attrition-related differences was generally limited. The descriptive statistics for age and other baseline characteristics reported above refer to the full T1 sample (*n* = 1,500), whereas descriptive statistics reported for the longitudinal analyses are based on the final analytic sample of 1,003 participants.

### Measures

Hand-gesture-dance participation (HGDP) was assessed using two self-reported indicators. First, participants reported their mode of participation, which was categorized into three levels: watching only, Privately imitating without recording or sharing, and Recording and posting the imitation on social media platforms. These categories were coded as 1, 2, and 3, respectively. Participants who did not watch or were unfamiliar with hand-gesture dance were excluded during the data collection stage. Second, participants reported their frequency of watching or imitating hand-gesture dance on a seven-point Likert scale. Previous research has conceptualized participation in terms of “participation intensity,” emphasizing the extent to which individuals engage in activities rather than simply whether they participate (Bult et al., 2010; Denault and Poulin, 2009). Following this approach, we operationalized hand-gesture-dance participation by combining participation mode and participation frequency. Specifically, the participation mode score was multiplied by the frequency score to generate a composite participation-intensity index ranging from 1 to 21, thereby capturing both the level and frequency of individuals' behavioral engagement with hand-gesture dance.

At T1, the index had a mean of 10.98 (SD = 4.983), compared with 9.45 (SD = 4.903) at T2. The distributions showed limited skewness (0.152 and 0.552, respectively; Supplementary Table S1). Participation mode showed moderate longitudinal stability, with 55.4%-78.0% of participants remaining in the same category across waves (Supplementary Table S2). The composite index also demonstrated moderate test-retest reliability (Spearman's ρ = 0.608, *p* < 0.001; ICC = 0.570, 95% CI [0.478, 0.643]; Supplementary Table S3). It was negatively associated with self-surveillance, interpersonal sensitivity, and anxiety at both waves (all *ps* ≤ 0.01; Supplementary Table S4), providing evidence of criterion-related associations in the theoretically expected directions. Sensitivity analyses using categorical participation mode and separate frequency measures yielded comparable results (Supplementary Table S5).

Self-surveillance (SS) was measured using the Objectified Body Consciousness Scale (OBCS) developed by McKinley and Hyde (1996), which was adapted for the present study. The adapted scale consisted of six items (e.g., When I watch or attempt hand-gesture dance movements, I pay attention to my body movements and appearance). Responses were rated on a seven-point Likert scale ranging from 1 (strongly disagree) to 7 (strongly agree), with higher scores indicating higher levels of self-surveillance. Confirmatory factor analyses indicated that the three-factor model showed better relative fit than the corresponding two-factor and one-factor alternatives at both waves. The standardized factor loadings for the six self-surveillance items ranged from 0.202 to 0.774 at T1 and from 0.227 to 0.788 at T2. The composite reliability (CR) values were 0.776 and 0.800, respectively, while the average variance extracted (AVE) values were 0.391 and 0.427. At the two time points, the Cronbach's α coefficients of the scale were 0.744 and 0.775, respectively.

Interpersonal sensitivity (IS) was measured using the interpersonal sensitivity dimension of the Symptom Checklist-90 (SCL-90) originally developed by Derogatis and colleagues (1976). The SCL-90 contains nine dimensions, and the present study adapted the eight items of the interpersonal sensitivity subscale (e.g., I am particularly sensitive to others' attitudes or reactions toward me on social media platforms). Responses were assessed using a seven-point Likert scale ranging from 1 (strongly disagree) to 7 (strongly agree), with higher scores indicating higher interpersonal sensitivity. The standardized factor loadings of the eight items ranged from 0.401 to 0.861 at T1 and from 0.459 to 0.885 at T2. The CR values were 0.913 and 0.924, respectively, and the AVE values were 0.574 and 0.609. At the two time points, the Cronbach's α coefficients of the scale were 0.912 and 0.920, respectively.

Anxiety (ANX) was also measured using the SCL-90 (Derogatis et al., 1976). The items were contextualized to capture anxiety experienced in relation to hand-gesture dance participation, social evaluation, and platform feedback. Accordingly, the resulting construct should be interpreted as context-specific anxiety rather than as a measure of general anxiety symptoms or clinical anxiety disorders. The anxiety subscale was adapted, resulting in a five-item measure (e.g., I feel nervous or afraid easily due to others' reactions, likes, or comments). Responses were assessed using a seven-point Likert scale ranging from 1 (strongly disagree) to 7 (strongly agree), with higher scores indicating higher levels of anxiety. The standardized factor loadings of the five items ranged from 0.660 to 0.854 at T1 and from 0.686 to 0.866 at T2. The CR values were 0.868 and 0.883, respectively, and the AVE values were 0.580 and 0.603. At the two time points, the Cronbach's α coefficients of the scale were 0.817 and 0.884, respectively.

For the subsequent cross-lagged panel analyses, observed composite scores were calculated for each construct by averaging the corresponding items at each measurement time point. The full wording of all adapted items is provided in the Supplementary Material S1. Confirmatory factor analyses conducted separately at T1 and T2 showed that the hypothesized three-factor model provided a better relative fit than the two-factor and one-factor alternatives (T1: Δχ^2^ = 114.76 and 1137.63; T2: Δχ^2^ = 134.66 and 1,402.50). Although the absolute fit of the three-factor model fell below the conventional thresholds at both waves (Supplementary Table S6), it showed consistently better relative fit than the two-factor and one-factor alternatives, supporting the retention of self-surveillance, interpersonal sensitivity, and anxiety as distinct constructs; this weaker absolute fit is consistent with the relatively low convergent validity observed for self-surveillance, as reported above. Detailed results are reported in Supplementary Table S6. Discriminant validity was additionally assessed using the heterotrait-monotrait ratio (HTMT). The HTMT values for self-surveillance and interpersonal sensitivity were 0.480 at T1 and 0.513 at T2, while those for self-surveillance and anxiety were 0.409 and 0.436, respectively, all below the conservative threshold of 0.85. In contrast, the HTMT values between interpersonal sensitivity and anxiety were relatively high (0.925 at T1 and 0.930 at T2), indicating substantial overlap between these two constructs. This overlap should therefore be considered when interpreting their distinction in the present context.

### Statistical analysis

Statistical analyses were conducted using SPSS 24.0. Categorical variables are presented as frequencies and percentages. For continuous variables, normality was assessed using the Kolmogorov-Smirnov test. Variables following a normal distribution are reported as mean ± standard deviation, whereas non-normally distributed variables are presented as median (Q1, Q3). Pearson correlation analyses were performed to examine associations between hand-gesture dance participation, self-surveillance, interpersonal sensitivity, and anxiety at both time points. A two-wave traditional cross-lagged panel model (CLPM) was estimated using Mplus 8.3 to examine the exploratory temporal associations among these variables. All constructs were represented by observed composite scores calculated from the corresponding questionnaire items rather than latent variables. Hand-gesture dance participation was likewise treated as an observed composite score. The models included autoregressive and cross-lagged paths between the two measurement occasions, with within-wave residual covariances among the T2 endogenous variables freely estimated. Maximum likelihood (ML) estimation was used, and standardized coefficients were reported using the STDYX standardization. No covariate effects were imposed on the T2 variables in the final model. Statistical significance was defined as *p* < 0.05. Associations with *p* = 0.050 were described as marginally significant and interpreted cautiously rather than as conventionally significant.

Harman's single-factor test was conducted separately for the items measured at T1 and T2 to assess the potential for common method variance. At T1, four factors had eigenvalues greater than 1, with the first factor explaining 40.663% of the total variance. At T2, three factors had eigenvalues greater than 1, with the first factor explaining 43.309% of the total variance. In both waves, the variance explained by the first factor was below the 50% threshold (Tang and Wen, 2020), indicating that a single factor did not account for the majority of the variance.

## Results

### Descriptive analysis

Table 1 presents the means, standard deviations, and correlations among the study variables. Hand-gesture dance participation was negatively correlated with self-surveillance, interpersonal sensitivity, and anxiety at both T1 and T2, with correlations ranging from −0.097 to 0.306 (*ps* < 0.01). These preliminary associations provide initial evidence of an inverse relationship between hand-gesture dance participation and the three psychological outcomes, which was further examined using longitudinal analyses.

**Table 1:** Descriptive statistics and correlations among study variables.

Variable	1	2	3	4	5	6	7	8	9	10	11
1. Gender	-										
2. Age	−0.054	-									
3. EDU	0.096^**^	−0.178^***^	-								
4. HGDP_T1	0.026	0.042	0.064^*^	-							
5. HGDP_T2	0.100^**^	0.022	0.116^***^	0.597^***^	-						
6. SS_T1	0.105^**^	−0.076^*^	0.088^**^	−0.166^***^	−0.169^***^	-					
7. SS_T2	0.136^***^	−0.120^***^	0.031	−0.102^**^	−0.097^**^	0.617^***^	-				
8. IS_T1	0.121^***^	−0.117^***^	−0.055	−0.235^***^	−0.294^***^	0.421^***^	0.377^***^	-			
9. IS_T2	0.130^***^	−0.143^***^	−0.03	−0.302^***^	−0.306^***^	0.356^***^	0.464^***^	0.750^***^	-		
10. ANX_T1	0.145^***^	−0.156^***^	−0.021	−0.219^***^	−0.242^***^	0.373^***^	0.337^***^	0.835^***^	0.666^***^	-	
11. ANX_T2	0.160^***^	−0.212^***^	−0.007	−0.281^***^	−0.276^***^	0.315^***^	0.406^***^	0.702^***^	0.849^***^	0.717^***^	-
M	1.66	32.60	5.02	10.98	9.45	4.67	4.72	3.59	3.60	2.90	2.94
SD	0.474	8.146	0.699	4.983	4.903	0.897	0.910	1.259	1.276	1.204	1.251

^*^p < 0.05; ^**^p < 0.01; ^***^p < 0.001 (two-tailed). EDU, education level; HGDP, hand-gesture dance participation; SS, self-surveillance; IS, interpersonal sensitivity; ANX, anxiety. *n* = 1003.

### Cross-lagged analysis

To examine the temporal associations among hand-gesture dance participation, self-surveillance, interpersonal sensitivity, and anxiety, six competing two-wave traditional cross-lagged panel models (CLPMs) were tested following the procedure proposed by Martens and Haase (2006). The model comparison was used to compare the relative fit of the six candidate models.

Specifically, Model 1 (M1) served as the baseline model and included only autoregressive paths among hand-gesture dance participation, self-surveillance, interpersonal sensitivity, and anxiety. Model 2 (M2) added cross-lagged paths from hand-gesture dance participation at T1 to self-surveillance, interpersonal sensitivity, and anxiety at T2. Model 3 (M3) added cross-lagged paths from self-surveillance at T1 to hand-gesture dance participation, interpersonal sensitivity, and anxiety at T2. Model 4 (M4) added cross-lagged paths from interpersonal sensitivity at T1 to hand-gesture dance participation, self-surveillance, and anxiety at T2. Model 5 (M5) added cross-lagged paths from anxiety at T1 to hand-gesture dance participation, self-surveillance, and interpersonal sensitivity at T2. Model 6 (M6) included all paths specified in M1 through M5.

Model fit was evaluated using χ^2^, degrees of freedom (df), comparative fit index (CFI), Tucker-Lewis index (TLI), root mean square error of approximation (RMSEA) with its 90% confidence interval, standardized root mean square residual (SRMR), Akaike information criterion (AIC), and Bayesian information criterion (BIC). To examine the temporal associations among hand-gesture dance participation, self-surveillance, interpersonal sensitivity, and anxiety, six competing two-wave traditional cross-lagged panel models (CLPMs) were tested following the procedure proposed by (Martens and Haase 2006). Model 1 (M1) served as the baseline model and included only autoregressive paths among the four constructs. Models 2–5 sequentially added cross-lagged paths originating from hand-gesture dance participation, self-surveillance, interpersonal sensitivity, and anxiety, respectively. Model 6 (M6) included all autoregressive and cross-lagged paths specified in the preceding models.

Model fit was evaluated using χ^2^, degrees of freedom (df), comparative fit index (CFI), Tucker-Lewis index (TLI), root mean square error of approximation (RMSEA) with its 90% confidence interval, standardized root mean square residual (SRMR), Akaike information criterion (AIC), and Bayesian information criterion (BIC). Among the candidate models, M6 showed the best fit according to the overall pattern of comparative fit indices and information criteria, with CFI = 0.958, TLI = 0.925, RMSEA = 0.075 (90% CI [0.065, 0.085]), SRMR = 0.071, AIC = 22,402.610, and BIC = 22,579.397. These absolute fit indices met conventional acceptability criteria (CFI and TLI > 0.90; RMSEA and SRMR < 0.08). The model fit indices for all six competing models are presented in Table 2. M6 was therefore retained as both the best-fitting model among the alternatives and an acceptably fitting model for examining the temporal associations among the four constructs.

**Table 2:** Model fit indices.

MODEL	*χ^2^*	df	*p*	CFI	TLI	RMSEA	90% CI	SRMR	AIC	BIC
M1	537.423	39	< 0.001	0.863	0.828	0.113	[0.105, 0.121]	0.111	22,737.486	22,855.344
M2	460.881	36	< 0.001	0.883	0.841	0.108	[0.100, 0.117]	0.105	22,666.944	22,799.534
M3	496.929	36	< 0.001	0.874	0.828	0.113	[0.104, 0.122]	0.108	22,702.991	22,835.581
M4	320.576	36	< 0.001	0.922	0.894	0.089	[0.080, 0.098]	0.080	22,526.639	22,659.229
M5	400.113	36	< 0.001	0.900	0.864	0.100	[0.092, 0.109]	0.083	22,606.176	22,738.766
M6	178.547	27	< 0.001	0.958	0.925	0.075	[0.065, 0.085]	0.071	22,402.610	22,579.397

χ^2^, chi-square; df, degrees of freedom; CFI, comparative fit index; TLI, Tucker-Lewis index; RMSEA, root mean square error of approximation; SRMR, standardized root mean square residual. Model fit is considered acceptable when CFI and TLI > 0.90 and RMSEA and SRMR < 0.08.

All coefficients reported below are STDYX standardized regression coefficients. The autoregressive paths were significant for all four constructs. Hand-gesture dance participation at T1 was positively associated with hand-gesture dance participation at T2 (β = 0.505, 95% CI [0.459, 0.551], *p* < 0.001). The results of Model 6 are presented in Figure 2. Self-surveillance at T1 was positively associated with self-surveillance at T2 (β = 0.539, 95% CI [0.490, 0.588], *p* < 0.001). Interpersonal sensitivity at T1 was positively associated with interpersonal sensitivity at T2 (β = 0.576, 95% CI [0.505, 0.648], *p* < 0.001), and anxiety at T1 was positively associated with anxiety at T2 (β = 0.420, 95% CI [0.347, 0.492], *p* < 0.001).

**Figure 2:**
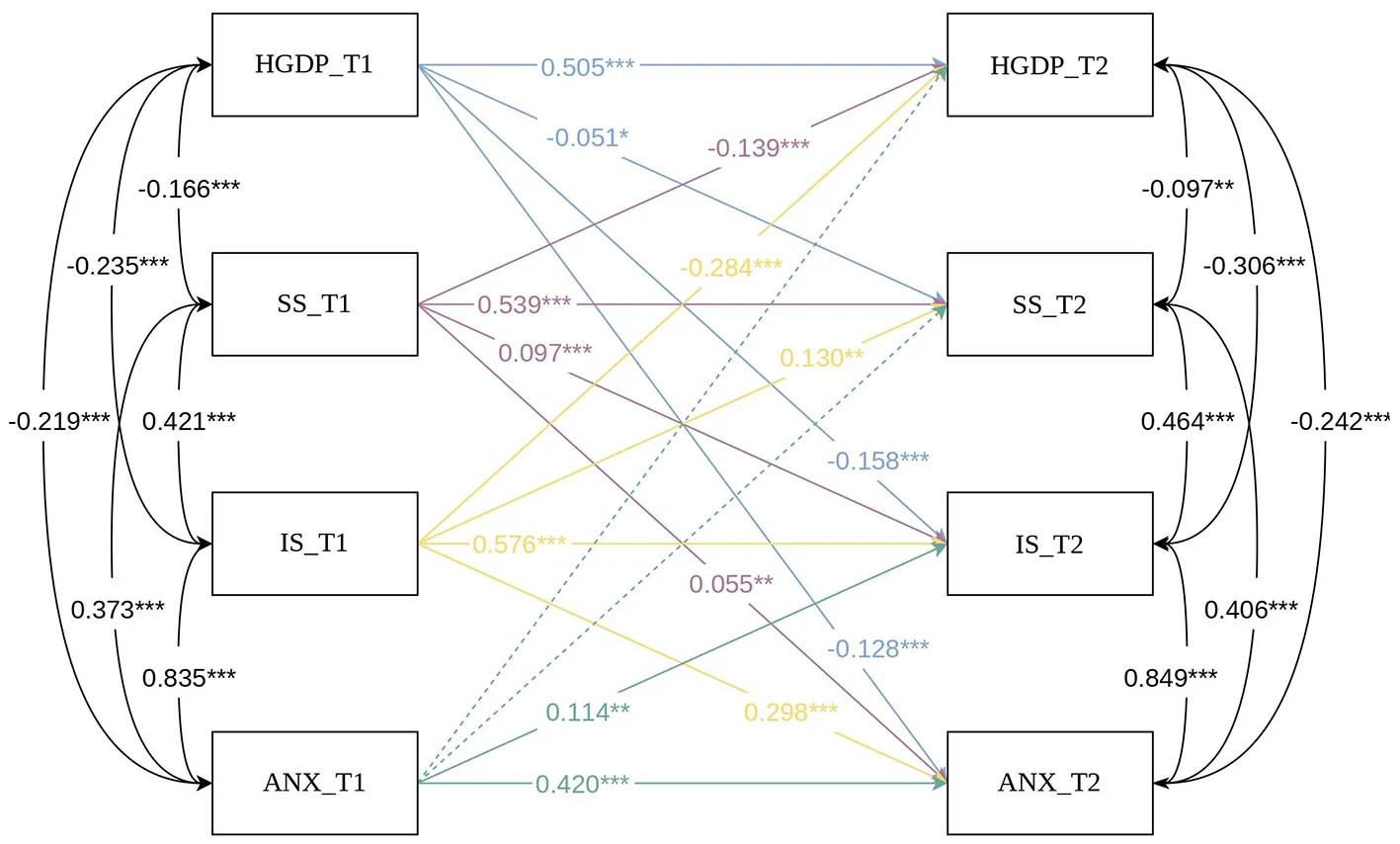
Results of model 6 (M6).

Regarding the cross-lagged associations predicting HGDP at T2, self-surveillance at T1 was positively associated with HGDP at T2 (β = 0.193, 95% CI [0.138, 0.247], *p* < 0.001), whereas interpersonal sensitivity at T1 was negatively associated with HGDP at T2 (β = −0.284, 95% CI [−0.373, −0.195], *p* < 0.001). Anxiety at T1 was not significantly associated with HGDP at T2 (β = 0.034, 95% CI [−0.053, 0.120], *p* = 0.446).

For self-surveillance at T2, HGDP at T1 showed a marginally significant negative association (β = −0.051, 95% CI approximately [−0.102, 0.000], *p* = 0.050). Interpersonal sensitivity at T1 was positively associated with self-surveillance at T2 (β = 0.130, 95% CI [0.040, 0.221], *p* = 0.005), whereas anxiety at T1 was not significantly associated with self-surveillance at T2 (β = 0.038, 95% CI [−0.049, 0.126], *p* = 0.391).

For interpersonal sensitivity at T2, HGDP at T1 was negatively associated with interpersonal sensitivity (β = −0.158, 95% CI [−0.201, −0.115], *p* < 0.001). Self-surveillance at T1 (β = 0.097, 95% CI [0.052, 0.143], *p* < 0.001) and anxiety at T1 (β = 0.114, 95% CI [0.042, 0.186], *p* = 0.002) were both positively associated with interpersonal sensitivity at T2.

For anxiety at T2, HGDP at T1 was negatively associated with anxiety (β = −0.128, 95% CI [−0.173, −0.084], *p* < 0.001). Self-surveillance at T1 (β = 0.055, 95% CI [0.007, 0.102], *p* = 0.024) and interpersonal sensitivity at T1 (β = 0.298, 95% CI [0.222, 0.375], *p* < 0.001) were positively associated with anxiety at T2.

Taken together, the results indicate substantial temporal stability in the four constructs and multiple cross-lagged associations across the one-1 interval. HGDP at T1 was associated with lower subsequent interpersonal sensitivity and anxiety and showed a marginally significant negative association with subsequent self-surveillance. The complete path estimates and hypothesis-testing results are presented in Table 3.

**Table 3:** Standardized cross-lagged path coefficients and hypothesis testing.

Dependent variable	Predictor	β	95% CI	*p*	Hypothesis decision
HGDP_T2	HGDP_T1	0.505	[0.459, 0.551]	< 0.001	Supported
	SS_T1	0.193	[0.138, 0.247]	< 0.001	Supported
	IS_T1	−0.284	[−0.373, −0.195]	< 0.001	Supported
	ANX_T1	0.034	[−0.053, 0.120]	0.446	-
SS_T2	SS_T1	0.539	[0.490, 0.588]	< 0.001	Supported
	HGDP_T1	−0.051	[−0.102, 0.000]	0.050	Marginally Supported
	IS_T1	0.13	[0.040, 0.221]	0.005	Supported
	ANX_T1	0.038	[−0.049, 0.126]	0.391	-
IS_T2	IS_T1	0.576	[0.505, 0.648]	< 0.001	Supported
	HGDP_T1	−0.158	[−0.201, −0.115]	< 0.001	Supported
	SS_T1	0.097	[0.052, 0.143]	< 0.001	Supported
	ANX_T1	0.114	[0.042, 0.186]	0.002	Supported
ANX_T2	ANX_T1	0.42	[0.347, 0.492]	< 0.001	Supported
	HGDP_T1	−0.128	[−0.173, −0.084]	< 0.001	Supported
	SS_T1	0.055	[0.007, 0.102]	0.024	Supported
	IS_T1	0.298	[0.222, 0.375]	< 0.001	Supported

Overall, H1-H6 received varying degrees of support. H1 was partially supported, as the associations between hand-gesture dance participation and self-surveillance were positive in one direction and marginally negative in the other. H2 was supported, with significant reciprocal associations between hand-gesture dance participation and interpersonal sensitivity. H3 was partially supported, as hand-gesture dance participation negatively predicted subsequent anxiety, whereas anxiety did not significantly predict subsequent participation. H4, H5, and H6 were supported, with significant reciprocal temporal associations between self-surveillance and interpersonal sensitivity, self-surveillance and anxiety, and interpersonal sensitivity and anxiety, respectively.

## Discussion

### Main findings and theoretical implications

The present study provides partial support for the proposed temporal associations among hand-gesture dance participation, self-surveillance, interpersonal sensitivity, and anxiety.

A notable finding of the present study is that hand-gesture dance participation was negatively associated with subsequent anxiety (β = −0.128, *p* < 0.001), indicating that greater engagement in hand-gesture dance was associated with lower subsequent levels of anxiety. This finding provides longitudinal evidence for the potential of hand-gesture dance as an everyday behavioral activity associated with lower anxiety. One possible interpretation is that the pleasurable and absorbing features of hand-gesture dance may be associated with reduced attention to worries. The synchronization of bodily movement with music may also facilitate physical and psychological relaxation by reducing accumulated tension and promoting a more positive emotional state. Unlike structured forms of exercise that may require considerable time, physical effort, or specific facilities, hand-gesture dance is characterized by simple movements and a low threshold for participation, making it readily accessible in everyday life. Its integration into short-form social media content further enables individuals to engage in the activity spontaneously and repeatedly, potentially allowing its emotion-regulatory benefits to be incorporated into daily routines. Moreover, the expressive and playful nature of hand-gesture dance may provide individuals with an opportunity to momentarily disengage from performance-oriented or stressful everyday experiences and experience greater enjoyment and emotional release. Taken together, these characteristics may provide one possible explanation for the observed negative temporal association between hand-gesture dance participation and subsequent anxiety. The present finding therefore extends our understanding of hand-gesture dance beyond its entertainment function and highlights its potential psychological value as an everyday activity associated with lower anxiety over time.

Within the overall structure of the cross-lagged model, the variables exhibited multidirectional longitudinal associations rather than a single unidirectional pathway. The most direct longitudinal associations involving hand-gesture dance participation were its negative cross-lagged associations with subsequent anxiety and self-surveillance. Specifically, higher hand-gesture dance participation at T1 was associated with lower anxiety at T2 and showed a marginally significant negative association with self-surveillance at T2., while self-surveillance, interpersonal sensitivity, and anxiety also showed several significant cross-lagged associations with one another. These findings provide preliminary evidence of a temporal association between hand-gesture dance participation and self-surveillance, while also highlighting the heterogeneity of psychological outcomes associated with different forms of social media engagement. Prior research on short-video participation has largely emphasized its positive associations with anxiety, attentional fragmentation, or emotional instability (Jain et al., 2025). In contrast, the present study observed a different pattern for a specific form of embodied participation. Studies on health-related video consumption further suggest that users engage in distinct consumption activities, including watching videos to meet informational, emotional, and entertainment needs, evaluating video credibility and interestingness, and using videos to achieve health-related goals (Liu and Zhang, 2024). This indicates that heterogeneous participation modes within short-video environments may correspond to distinct psychological experiences.

Further, the longitudinal design provides a stronger basis for examining temporal associations between hand-gesture dance participation and psychological variables than a cross-sectional design. The longitudinal associations observed in the model suggest that the relationship between hand-gesture dance participation and self-surveillance may unfold over time rather than being limited to concurrent associations. From this perspective, repeated engagement may be particularly relevant to the psychological significance of embodied participation, as behavioral routines and repeated physical activities can gradually become integrated into individuals' everyday experiences. This suggests that the psychological significance of hand-gesture dance may extend beyond transient emotional responses and may be shaped by continued engagement over time. Prior longitudinal evidence also supports this interpretation, showing that physical activity not only correlates with psychological variables but also predicts later psychological changes through cross-time pathways, demonstrating clear lagged effects (Wu et al., 2025). From a theoretical perspective, this cumulative pattern aligns with research on habit formation and psychological adaptation in the physical activity literature. Behavioral stability in health-related practices depends on repeated engagement and habit formation processes (Singh et al., 2024), while physical activity, as a routine behavior, gradually becomes automated through repetition (Ma et al., 2023). The 1-month longitudinal interval allowed the present study to examine whether participation at T1 was associated with psychological outcomes measured at T2. The two-wave design focused on temporal associations across the 1-month interval, while leaving open whether these associations develop gradually, occur immediately, or reflect more stable between-person differences. The present findings underscore the importance of longitudinal perspectives for understanding embodied participation behaviors, which may not be fully captured by cross-sectional designs.

Notably, the results also suggest that hand-gesture dance participation is functionally closer to traditional forms of physical exercise or dance activity than to mere information consumption or general social media use. Meanwhile, hand-gesture dance does not exist as a traditional physical activity detached from the digital media environment; rather, it represents a new form of embodied participation emerging through social media platforms. Although it is embedded within short-video platforms, its core features lie not in media technology per se but in embodied actions performed by participants, including movement execution, rhythm coordination, and sustained practice (Yu et al., 2024). From this perspective, the platform functions primarily as a mediating environment, whereas the observed psychological associations may partly reflect the embodied nature of the activity itself, rather than being attributable solely to the characteristics of the digital platform. Existing research on social media and mental health has predominantly focused on media exposure, informational content, and online interaction (Gu et al., 2023; Shakya and Christakis, 2017; Shi et al., 2024), while paying relatively less attention to the action-based nature of participation itself. The present findings suggest that the intrinsic properties of participatory behavior, rather than the medium or channel alone, may be relevant to its psychological associations. Therefore, hand-gesture dance participation should not be conceptualized solely as a digital media phenomenon but should also be understood within frameworks of physical activity and embodied experience.

To explore potential explanations for the observed association between hand-gesture dance participation and self-surveillance, an embodied engagement perspective may provide a useful theoretical interpretation. Hand-gesture dance participation showed a small, marginally significant negative association with subsequent self-surveillance, while self-surveillance was positively associated with subsequent interpersonal sensitivity. From an embodied engagement perspective, this process may be driven by immersion and flow experience. Flow theory posits that high levels of task engagement and action execution consume cognitive resources, thereby producing a state of loss of self-consciousness (Csikszentmihalyi, 1991). Compared with text posting, image browsing, or information-based interaction, hand-gesture dance participation requires sustained attention to movement execution, rhythm tracking, and bodily coordination. During this process, cognitive resources are allocated primarily to task execution rather than self-image monitoring, external evaluation, or social comparison. Prior dance research indicates that individuals immersed in dance activities often experience reduced self-focus and a shift of attention from self-monitoring to bodily movement and immediate experience, thereby achieving strong immersion and flow states (Koch and Fischman, 2011). Although hand-gesture dance is relatively simple in form, it retains essential features of dance-based embodied activity. Therefore, the embodied nature of dance may represent one possible explanation for the observed association between hand-gesture dance participation and self-surveillance.

In addition, the significant cross-lagged associations between self-surveillance and interpersonal sensitivity provide a potential basis for considering how changes in self-focused attention may relate to interpersonal sensitivity, which is characterized by excessive concern and heightened reactivity to others' evaluations, social feedback, and interpersonal cues (Fenigstein et al., 1975). When individuals engage in high levels of self-surveillance, attention is primarily directed toward self-performance and anticipated external evaluation, which reinforces vigilance regarding social positioning and image management. This sustained self-monitoring increases concerns about social evaluation and, in turn, heightens interpersonal sensitivity. When hand-gesture dance participation shifts attention toward task engagement and embodied experience, cognitive resources are redirected away from self-monitoring, thereby reducing excessive concern about external evaluation (Ingram, 1990). As self-surveillance decreases, sensitivity to potential interpersonal threats also diminishes, leading to lower interpersonal sensitivity. This further suggests that immersion and attentional shifts associated with embodied action may represent one possible psychological process underlying the association between hand-gesture dance participation and psychological outcomes.

Based on the above results, this study offers further theoretical implications. On the one hand, the findings provide empirical evidence for re-examining and refining self-surveillance theory in digital media contexts. Within traditional theoretical frameworks, self-surveillance is typically conceptualized as a relatively stable individual trait, reflecting the extent to which individuals consistently attend to their own states and others' evaluations. Existing research generally suggests that self-surveillance primarily emerges in contexts characterized by heightened social evaluation and self-presentation demands and is closely associated with individuals' sensitivity to external observation and evaluation (Kawamoto, 2021). However, the present study provides preliminary evidence that self-surveillance may show temporal variability in social media contexts and may be temporally associated with embodied media engagement, while hand-gesture dance participation was also directly associated with subsequent anxiety, suggesting that digital participatory practices may warrant further consideration as contextual factors in research on both self-surveillance and psychological wellbeing. On the other hand, this study extends the understanding of the psychological consequences of embodied social media use from a longitudinal perspective. While prior research has primarily focused on immediate effects or cross-sectional associations, the lagged associations observed in the cross-lagged model suggest that embodied participation may be associated with subsequent levels of anxiety and social cognition over time, although their temporal ordering, stability, and potential explanations require further investigation.

Overall, this study provides several implications for understanding the relationship between social media participation and mental health. First, social media participation is not a homogeneous construct, and different forms of engagement may yield substantially different psychological outcomes. Future research should move beyond broad categorizations such as use vs. non-use or high vs. low frequency and instead focus on the specific characteristics of participatory behavior, including its action-based nature, embodied involvement, and contextual conditions, as these characteristics may be differentially associated with anxiety and other psychological outcomes. Second, embodied participatory activities within social media environments warrant further investigation as a distinct form of digital engagement. Activities such as dance, singing, or other forms of embodied engagement share common features of active bodily involvement and sustained attention, which may facilitate immersion and reduce excessive self-focus, potentially contributing to more positive psychological experiences. Even relatively simple and easily imitable forms such as the hand-gesture dance may provide opportunities for flow experience and reduced self-surveillance. By directing attention toward movement execution and embodied experience, such participation may be associated with reduced self-focused attention and lower anxiety, while changes in self-focused attention may also be relevant to interpersonal sensitivity. In the context of increasingly pervasive digital media environments, encouraging users to engage in more creative, action-oriented, and embodied interactive activities may provide a useful direction for understanding how different modes of social media engagement are associated with psychological wellbeing.

### Limitations and future directions

Several limitations should be considered when interpreting the findings. First, the two-wave longitudinal design limits the ability to establish the temporal ordering required to test the proposed sequential process among hand-gesture dance participation, self-surveillance, interpersonal sensitivity, and anxiety. Because all constructs were measured at the same two time points, their cross-lagged associations occurred over the same T1-T2 interval. Therefore, the present findings cannot establish whether changes in one construct subsequently lead to changes in another. Future studies should employ at least three additional appropriately spaced measurement waves, depending on the hypothesized temporal structure, to directly examine the proposed temporal ordering. In addition, because the present analysis used a traditional two-wave CLPM, the observed cross-lagged associations may partly reflect stable between-person differences rather than within-person changes. Accordingly, these coefficients should be interpreted as temporal associations rather than within-person or causal effects. Future research could use random-intercept cross-lagged panel models or other longitudinal approaches that explicitly separate between-person and within-person processes.

Second, the sampling procedure may limit the generalizability of the findings. Participants were recruited through nationwide open recruitment rather than probability-based, quota-based, or stratified sampling, and eligibility required familiarity with and prior exposure to hand-gesture dance content. The resulting sample therefore represents adults who had already encountered hand-gesture dance and does not permit direct comparison with individuals who had never been exposed to such content. Familiarity with hand-gesture dance may also be associated with age, social-media use intensity, and psychological characteristics, potentially introducing selection bias. Future research should recruit participants with different levels of exposure, including individuals who have never encountered or participated in hand-gesture dance, and should employ probability-based or stratified sampling where feasible.

Third, the study relied primarily on self-reported measures, which may be subject to recall bias and social desirability effects. Self-reported participation may not accurately capture actual behavioral engagement with short-video content. Future research should integrate objective behavioral trace data, such as platform usage time, viewing records, likes, comments, and posting behavior, where ethically and practically feasible. Combining self-report measures with behavioral data would improve the ecological validity and measurement accuracy of research on embodied social media participation. In addition, the anxiety measure was substantially adapted to the hand-gesture-dance and social-media context. The items primarily captured anxiety related to participation, social evaluation, and platform feedback rather than general anxiety symptoms. The present findings should therefore not be generalized directly to clinical anxiety or anxiety disorders. Future research should include validated measures of general anxiety alongside context-specific measures to examine the extent to which these constructs overlap.

Fourth, although Harman's single-factor test did not indicate that a single factor accounted for the majority of the variance, this procedure cannot rule out common method variance. The 1-month temporal separation between the two waves may have reduced some shared-method effects, but the use of the same respondents, self-report measures, similar response formats, and related item contexts may still have introduced common method variance. Because a marker variable was not included and a common-method-factor model could not be tested with the available data, residual common method bias cannot be completely excluded. Future studies should incorporate procedural and statistical remedies, including multiple data sources, marker variables, and comparisons between theoretical measurement models and common-method-factor models.

Finally, potential confounding factors were not comprehensively assessed or controlled. Although demographic characteristics such as gender and age may be associated with both psychological outcomes and hand-gesture dance participation, they were not included as covariates in the final analyses. Other potentially relevant factors, including general short-video use, offline physical activity or dance participation, previous experience with posting videos, social feedback such as comments and likes, socioeconomic status, education, and pre-existing psychological characteristics, were also not comprehensively measured. Consequently, the observed temporal associations cannot be interpreted as effects independent of these potential confounding factors. Future research should prespecify covariates based on theoretical and causal considerations and collect more comprehensive information on physical activity, media-use patterns, social feedback, and relevant psychological characteristics to obtain more rigorous estimates of the associations between hand-gesture dance participation and psychological outcomes.

## Conclusion

This study provides longitudinal evidence that hand-gesture dance participation is temporally associated with psychological wellbeing and self-related cognition in distinct ways. Specifically, greater hand-gesture dance participation at T1 was associated with lower anxiety at T2 and showed a marginally significant negative association with self-surveillance at T2, indicating that this form of embodied social media participation may be associated with more favorable psychological outcomes over time. At the same time, the significant cross-lagged associations among self-surveillance, interpersonal sensitivity, and anxiety indicate that these psychological variables are dynamically interconnected rather than operating through a simple unidirectional pathway. These findings suggest that the psychological significance of social media participation may depend substantially on how individuals engage with content and, in particular, may vary according to participation mode and level of behavioral involvement. The present findings also highlight the value of examining passive viewing and active embodied participation separately in future research.

The findings also contribute to a more differentiated understanding of social media use and mental health. Hand-gesture dance is embedded within short-video platforms, but its defining characteristics involve bodily movement, rhythm coordination, imitation, and active participation. Its psychological associations may therefore partly reflect the embodied nature of the activity rather than exposure to digital media alone. The negative longitudinal association between hand-gesture dance participation and self-surveillance is consistent with the possibility that embodied engagement redirects attention from self-monitoring toward movement execution and immediate experience. Given the two-wave design, this interpretation is best understood as a theoretically informed explanation of the observed association, while the temporal ordering of a sequential process and the distinction between within-person effects and stable between-person differences warrant further investigation.

Overall, the present study highlights the importance of distinguishing different modes of social media participation when examining their psychological implications. Rather than treating social media use as a homogeneous exposure, future research should consider the behavioral and embodied characteristics of specific forms of participation. Activities that combine digital engagement with physical movement and active expression may represent an important but underexamined form of social media use. Further longitudinal and intensive repeated-measures research is needed to determine whether sustained embodied participation is associated with changes in self-surveillance, interpersonal sensitivity, and anxiety and to clarify the psychological mechanisms underlying these temporal associations.

## Data Availability

The raw data supporting the conclusions of this article will be made available by the authors, without undue reservation.
