# Supplementary material for: Does imitating the “hand-gesture dance” on social media reduce anxiety? A two-wave cross-lagged panel analysis on digital embodied practices
Source: Front Psychol. 2026 Oct 14;17:1934635. doi: 10.3389/fpsyg.2026.1934635 (PMC13713705; doi:10.3389/fpsyg.2026.1934635)
Supplement: Supplementary file 1 [file Supplementary_File_1.docx]

Supplementary Material

# 1 Supplementary Material

Supplementary Material S1. Survey Questionnaire

Below are the measurement items administered in the longitudinal survey. The informed-consent form presented here is the T2 follow-up version. The substantive measurement items were administered at both waves using the same response format.

**知情同意书**

尊敬的调查参与者：

您好！感谢您在第一次问卷填写中的支持。本研究为追踪调查，您目前参与的是第二轮问卷填写，用于帮助我们进一步了解数据在时间维度上的变化。本问卷大约需要您 10 分钟完成。

在填写问卷前，请您确认以下内容：

(1) 您此刻是否处于安静、不被打扰的环境中？

(2) 您是否已经完成过第一轮问卷，并愿意继续参与本次追踪填写？

我们将继续对您的个人信息严格保密，所有数据仅用于学术研究，并在分析前进行匿名化处理，不会对您造成任何影响。部分研究结果可能以论文或报告形式呈现，但不会包含任何可识别您身份的信息。

您仍然可以在填写过程中随时退出，本研究不会因此给您带来任何不利后果。

在充分了解本研究目的和可能影响后，如您同意参与第二轮调查，请勾选下方选项后继续作答；如您不同意，请关闭本页面。

感谢您再次支持本研究！

○ 我已认真阅读并确认参与

**Informed Consent Form**

Dear Participant,

Thank you for your support during the first round of our survey. This study is a longitudinal investigation; you are currently participating in the second round, which aims to examine temporal dynamics in the data. Completing this questionnaire will take approximately 10 minutes.

Before proceeding, please confirm the following:

(1) You are currently in a quiet, undisturbed environment.

(2) You have completed the first-round survey and agree to participate in this follow-up round.

Your personal data will remain strictly confidential. All information will be used exclusively for academic research and anonymized prior to statistical analysis. Findings may be presented in academic publications or reports, but no personally identifiable information will be disclosed.

Your participation is entirely voluntary, and you may withdraw at any time without penalty or adverse consequences.

By selecting the option below, you indicate that you have read and understood the purpose and implications of this study and agree to participate in the second round. If you do not wish to participate, please close this page.

Thank you again for your valuable contribution to this research.

○ I have read and understood the information above and agree to participate.

| **人口统计学变量** | **Demographic Variables** |
| --- | --- |
| 性别  ○ 男  ○ 女 | Gender  ○ Male  ○ Female |
| 请填写您的出生年份（例：1998） | Please enter your birth year (e.g., 1998) |
| 请选择您的最高学历  ○ 小学及以下  ○ 初中  ○ 普高/中专/技校/职高  ○ 大专  ○ 本科  ○ 硕士  ○ 博士及以上 | Please select your highest level of education  ○ Primary school or below  ○ Junior high school  ○ Senior high school / Secondary vocational school / Technical school / Vocational high school  ○ Associate degree  ○ Bachelor's degree  ○ Master's degree  ○ Doctoral degree or above |
| 请问您每月收入是多少（人民币）  ○ 5000及以下  ○ 5001-10000  ○ 10001-20000  ○ 20001-30000  ○ 30000以上 | What is your monthly income (in RMB)?  ○ ≤ 5,000  ○ 5,001–10,000  ○ 10,001–20,000  ○ 20,001–30,000  ○ > 30,000 |
| **手势舞参与** | **Hand-gesture-dance participation(HGDP)** |
| 过去一个月，您在日常生活通常是如何参与手势舞？  ○ 仅观看  ○ 在私下模仿，但不拍摄或分享  ○ 会拍摄自己的手势舞并发布在社交平台  ○ 完全不了解，也不观看 | Over the past month, how have you typically engaged with gesture dance videos in your daily life?  ○ Watching only  ○ Privately imitating without recording or sharing  ○ Recording and posting the imitation on social media platforms  ○ Unfamiliar with and do not watch them |
| 过去一个月，您日常观看/模仿/拍摄手势舞手势舞的频率是  几乎不 1 - 2 - 3 - 4 - 5 - 6 - 7 经常 | Over the past month, what is your frequency of watching/ practicing/ recording gesture dance videos in your daily life?  (Rarely) 1 - 2 - 3 - 4 - 5 - 6 - 7 (Very Frequently) |
| **自我审视** | **Self-surveillance(SS)** |
| 当我观看或尝试手势舞动作时，我会注意自己动作和外貌的状态。 | When watching or attempting gesture dance movements, I pay attention to my actions and physical appearance. |
| 在观看或模仿手势舞视频时，我会担心自己的动作或外貌是否被他人评价。 | When watching or imitating gesture dance videos, I worry about how my movements or appearance will be evaluated by others. |
| 与动作或外貌相比，我更关注自己身体的感觉（如舒适度、愉悦）。 | I am more concerned with how my body feels (e.g., comfort, pleasure) than with how my movements or appearance look. |
| 当观看或尝试手势舞动作时，我会将自己的动作或状态与他人在视频中的表现进行比较。 | When watching or attempting gesture dance movements, I compare my actions or appearance with those of others in the videos. |
| 我会关注自己在观看或参与手势舞时的穿着或外观是否得体或好看。 | I pay attention to whether my clothing or overall appearance looks appropriate or good when watching or participating in gesture dances. |
| 我会注意自己在他人（线上或线下）眼中呈现的动作或外貌。 | I am conscious of how my movements or appearance look to others (either online or offline). |
| **人际关系** | **Interpersonal sensitivity(IS)** |
| 当我看到别人拍摄或展示手势舞时，会不自觉地与他们比较。 | Feeling inclined to compare myself with others when seeing them record or showcase hand-gesture-dances. |
| 与他人一起讨论或模仿手势舞时，我会感到有些害羞或紧张。 | Feeling shy or uneasy when discussing or practicing hand-gesture-dance with others. |
| 当别人对我的短视频或舞蹈表现发表评论时，我的情绪容易受到影响。 | Having my feelings easily affected when others comment on my short videos or hand-gesture-dance performance. |
| 我常觉得别人并不理解我在拍摄或喜欢手势舞的原因。 | Feeling that people do not understand why I record or enjoy hand-gesture-dance. |
| 当别人表现得更好或更受欢迎时，我会觉得自己不如他们。 | Feeling inferior to others when they perform better or gain more popularity with their hand-gesture-dance. |
| 我担心别人会因为我拍摄或模仿手势舞而对我有负面看法。 | Feeling worried that people may hold negative views of me because I record or imitate hand-gesture-dance. |
| 当别人注视我或谈论我的视频时，我会感到不自在。 | Feeling uneasy when people are watching me or talking about my hand-gesture-dance videos. |
| 我对他人在平台上对我的态度或反应特别敏感。 | Feeling overly sensitive to others' attitudes or reactions toward me on the platform regarding my hand-gesture-dance. |
| **焦虑** | **Anxiety(ANX)** |
| 当我观看或参与手势舞时，会感到紧张或不自在。 | Feeling tense, nervous, or uneasy when watching or participating in hand-gesture-dance. |
| 我担心别人可能会评价我的手势舞水平或表现。 | Feeling worried or anxious that others might evaluate my hand-gesture-dance performance or skill level. |
| 当看到别人表现很好或受到关注时，我会感到焦虑或不安。 | Feeling anxious or unsettled when seeing others perform well or gain attention through hand-gesture-dance. |
| 我在观看或参与手势舞时，容易感到心神不宁或坐立不安。 | Feeling restless or unable to sit still when watching or participating in hand-gesture-dance. |
| 我容易因为他人的反应、点赞或评论而感到害怕或紧张。 | Feeling fearful or nervous about others' reactions, likes, or comments regarding my hand-gesture-dance content. |

# 2 Supplementary Tables

**Supplementary Table S1.** Descriptive Statistics of Hand-Gesture Dance Participation at T1 and T2

| **Measure** | **T1** | **T2** |
| --- | --- | --- |
| Participation mode, n (%) | | |
| Watching only | 173 (17.2%) | 310 (30.9%) |
| Private imitation | 451 (45.0%) | 437 (43.6%) |
| Recording/posting | 379 (37.8%) | 256 (25.5%) |
|  |  |  |
| Participation frequency | | |
| M (SD) | 4.83 (1.122) | 4.72 (1.155) |
| Median | 5 | 5 |
| Range | 1-7 | 1-7 |
| Skewness | -0.505 | -0.524 |
|  |  |  |
| Composite participation index | | |
| M (SD) | 10.98 (4.983) | 9.45 (4.903) |
| Median | 10 | 10 |
| Range | 2-21 | 1-21 |
| Skewness | 0.152 | 0.552 |

*Note.* Descriptive statistics at both T1 and T2 are based on the final longitudinal analytic sample of participants who completed both waves (n = 1003), rather than the full T1 baseline sample (n = 1500).

**Supplementary Table S2.** Cross-Wave Transition in Hand-Gesture Dance Participation Modes from T1 to T2

| **T1 \ T2** | **Watching only** | **Private imitation** | **Recording/posting** | **Total** |
| --- | --- | --- | --- | --- |
| Watching only | 135 (78.0%) | 31 (17.9%) | 7 (4.0%) | 173 |
| Private imitation | 128 (28.4%) | 284 (63.0%) | 39 (8.6%) | 451 |
| Recording/posting | 47 (12.4%) | 122 (32.2%) | 210 (55.4%) | 379 |
| Total | 310 | 437 | 256 | 1003 |

**Supplementary Table S3.** Test–Retest Reliability of the Composite Participation Index Across T1 and T2

| **Index** | **Estimate** | **95% CI** |
| --- | --- | --- |
| Pearson correlation | 0.597*** | - |
| Spearman correlation | 0.608*** | - |
| ICC, single measure | 0.570*** | [0.478, 0.643] |

**Supplementary Table S4.** Spearman Correlations Between Hand-Gesture Dance Participation and T1 and T2 Outcomes

| **Analysis** | **T1** | **T2** |
| --- | --- | --- |
| Participation → Self-surveillance | *ρ* = -0.160*** | *ρ* = -0.103** |
| Participation → Interpersonal sensitivity | *ρ* = -0.248*** | *ρ* = -0.305*** |
| Participation → Anxiety | *ρ* = -0.238*** | *ρ* = -0.288*** |

**Supplementary Table S5.** Sensitivity Analyses of Alternative Hand-Gesture Dance Participation Indicators Predicting T2 Outcomes

| **T1 predictor** | **Self-surveillance T2** | **Interpersonal sensitivity T2** | **Anxiety T2** |
| --- | --- | --- | --- |
| Private imitation | 0.043 (*p* = 0.239) | -0.013 (*p* = 0.677) | -0.047 (*p* = 0.143) |
| Recording/posting | 0.045 (*p* = 0.254) | -0.172 (*p* < 0.001) | -0.136 (*p* < 0.001) |
| Participation frequency | 0.001 (*p* = 0.968) | -0.029 (*p* = 0.238) | -0.074 (*p* = 0.004) |

**Supplementary Table S6.** Comparison of Alternative Measurement Models at T1 and T2

| **Time** | **Measurement model** | ***χ*²** | **df** | **CFI** | **TLI** | **RMSEA** | **SRMR** | **Δ*χ*²** | **Δdf** |
| --- | --- | --- | --- | --- | --- | --- | --- | --- | --- |
| T1 | Three-factor model | 2693.49 | 149 | 0.845 | 0.822 | 0.111 | 0.146 | - | - |
| T1 | Two-factor model | 2808.25 | 151 | 0.838 | 0.816 | 0.113 | 0.146 | 114.76 | 2 |
| T1 | One-factor model | 3831.12 | 152 | 0.775 | 0.747 | 0.132 | 0.120 | 1137.63 | 3 |
| T2 | Three-factor model | 2477.62 | 149 | 0.870 | 0.850 | 0.106 | 0.162 | - | - |
| T2 | Two-factor model | 2612.28 | 151 | 0.862 | 0.844 | 0.108 | 0.162 | 134.66 | 2 |
| T2 | One-factor model | 3880.12 | 152 | 0.791 | 0.765 | 0.133 | 0.127 | 1402.50 | 3 |
